# Clinical and genomic assessment of PD-L1 SP142 expression in triple-negative breast cancer

**DOI:** 10.1007/s10549-021-06193-9

**Published:** 2021-03-26

**Authors:** Sung Gwe Ahn, Seon-Kyu Kim, Jonathan H. Shepherd, Yoon Jin Cha, Soong June Bae, Chungyeul Kim, Joon Jeong, Charles M. Perou

**Affiliations:** 1 Department of Surgery, Gangnam Severance Hospital, Yonsei University College of Medicine, 712 Eon-juro, Gangnam-gu, Seoul, Republic of Korea; 2 Personalized Genomic Medicine Research Center, Korea Research Institute of Bioscience and Biotechnology (KRIBB),, Daejeon, Korea; 3 Lineberger Comprehensive Cancer Center, University of North Carolina, Chapel Hill, NC 27599 USA; 4 Department of Genetics, University of North Carolina, Chapel Hill, NC USA; 5 Department of Pathology, Gangnam Severance Hospital, Yonsei University College of Medicine, Seoul, Republic of Korea; 6 Department of Pathology, Guro Hospital, Korea University College of Medicine, Seoul, Korea

**Keywords:** Triple-negative breast cancer, SP142 PD-L1, Tumor-infiltrating lymphocytes, Immune-check point inhibitors

## Abstract

**Purpose:**

The SP142 PD-L1 assay is a companion diagnostic for atezolizumab in metastatic triple-negative breast cancer (TNBC). We strove to understand the biological, genomic, and clinical characteristics associated with SP142 PD-L1 positivity in TNBC patients.

**Methods:**

Using 149 TNBC formalin-fixed paraffin-embedded tumor samples, tissue microarray (TMA) and gene expression microarrays were performed in parallel. The VENTANA SP142 assay was used to identify PD-L1 expression from TMA slides. We next generated a gene signature reflective of SP142 status and evaluated signature distribution according to TNBCtype and PAM50 subtypes. A SP142 gene expression signature was identified and was biologically and clinically evaluated on the TNBCs of TCGA, other cohorts, and on other malignancies treated with immune checkpoint inhibitors (ICI).

**Results:**

Using SP142, 28.9% of samples were PD-L1 protein positive. The SP142 PD-L1-positive TNBC had higher CD8+ T cell percentage, stromal tumor-infiltrating lymphocyte levels, and higher rate of the immunomodulatory TNBCtype compared to PD-L1-negative samples. The recurrence-free survival was prolonged in PD-L1-positive TNBC. The SP142-guided gene expression signature consisted of 94 immune-related genes. The SP142 signature was associated with a higher pathologic complete response rate and better survival in multiple TNBC cohorts. In the TNBC of TCGA, this signature was correlated with lymphocyte-infiltrating signature scores, but not with tumor mutational burden or total neoantigen count. In other malignancies treated with ICIs, the SP142 genomic signature was associated with improved response and survival.

**Conclusions:**

We provide multi-faceted evidence that SP142 PDL1-positive TNBC have immuno-genomic features characterized as highly lymphocyte-infiltrated and a relatively favorable survival.

**Supplementary Information:**

The online version contains supplementary material available at 10.1007/s10549-021-06193-9.

## Introduction

The prognosis of triple-negative breast cancer (TNBC) patients remains inferior to other clinical subtypes of breast cancers, in part because it lacks ER, PgR, and HER2, which can be targeted by endocrine therapy or anti-HER2 therapy [1]. Recent studies have revealed that TNBC has more immunogenomic features including increased tumor-infiltrating lymphocytes (TILs), increased expression of immune-related molecules and signatures, and higher tumor mutational burden (TMB), amongst mammary malignancies [2, 3]. Therefore, targeting programmed death 1 (PD-1) or its ligand (PD-L1), in combination with chemotherapy, is an emerging approach to offer clinical benefits in both early and advanced TNBC [4–9].

Among recent breast cancer trials with immune targeting drugs, the IMPASSION-130 trial demonstrated that the anti-PD-L1 monoclonal antibody atezolizumab plus nab-paclitaxel prolonged progression-free survival (PFS) in patients with metastatic TNBC [5]. Furthermore, the researchers showed that overall survival (OS) was improved by adding atezolizumab in the PD-L1-positive subgroup, which was defined by PD-L1 expression on immune cells using the SP142 antibody-based immunohistochemistry assay. Based on these findings, the Food and Drug Administration approved the use of atezolizumab for patients with PD-L1-positive metastatic TNBC [10], with the SP142 PD-L1 assay as a companion diagnostic. In addition, a clinical trial conducted in patients with early TNBC showed that PD-L1-positivity was a strong predictor for pathological complete response (pCR) [11].

These findings led us to investigate the clinical and genomic characteristics associated with SP142 PD-L1+ TNBC, with the hypothesis that PD-L1 protein expression might be associated with better survival outcome in the absence of immunotherapies. In this study, we evaluated PD-L1 expression by SP142 assay on TNBC tissue microarray (TMA), investigated clinicopathological features of PD-L1+ cases, and performed genomic analyses to develop a SP142 gene expression signature. Clinical and genomic aspects of this signature were further assessed in The Cancer Genome Atlas (TCGA) [12] and other TNBC cohorts. Finally, we tested the SP142 signature in the cohorts of other malignancies treated with immune-check point inhibitors (ICIs).

## Methods

### Ethics statement

The institutional review board (IRB) of Gangnam Severance Hospital, Yonsei University, Seoul, Korea, approved the study in accordance with good clinical practice guidelines and the Declaration of Helsinki (local IRB approval number: 3-2013-0268). The need for informed consent was waived because of the retrospective design.

### Patients and tumor samples

Formalin-fixed paraffin-embedded (FFPE) TNBC tumor samples were selected from the database of breast cancer patients treated between January 1999 and December 2014 at Gangnam Severance Hospital, Yonsei University Medical College, Seoul, Korea. Tumor cellularity was defined as the percentage of invasive tumor nuclei and was over 80% in all breast cancer specimens. Exclusion criteria for patient samples included: samples only exhibiting in situ carcinoma of the breast, bilateral breast cancers, as well as non-epithelial origin breast cancer, such as phyllodes tumor, sarcoma, or lymphoma.

TNBC was defined by the lack of ER, PR and HER2 based on immunohistochemical (IHC) stain. For routine IHC studies, ER (1:100 clone 6F11; Novocastra, Newcastle upon Tyne, UK), PR (clone 16; Novocastra), HER2 (4B5 rabbit monoclonal antibody; Ventana Medical Systems, Tucson, AZ, USA), and Ki-67 (MIB-1; Dako, Glostrup, Denmark) were stained using formalin-fixed paraffin-embedded tissue sections as previously described [13, 14]. ER positivity and PR expression on immunohistochemistry were defined according to the modified Allred system. In our study, ER and PR positivity were defined as an Allred score ≥ 3. The HER2 status was considered positive with a score of 3+ and negative with a score of 0 or 1+ [15]. Tumors with a score of 2+ were sent for fluorescent in situ hybridization analysis according to the protocol given by the supplier (PathVysion kit; Vysis, Downers Grove, IL, USA or HER2 inform; Ventana). A total of 149 TNBC subjects were investigated in our study.

### Tissue microarray, SP142 PD-L1 assay, and other studies

TMA paraffin blocks were generated as previously described using an Accu Max Array tissue-arraying instrument (Petagen, Inc., Seoul, Korea) [16]. In each samples, a single core was punched in the area showing invasive tumor nuclei was comprised ≥ 80% to construct the TMA slides. PD-L1 expression, stromal TILs, and CD8 percentage were assessed from TMA slides.

PD-L1 expression was determined using VENTANA PD-L1 SP142 assay, which is a qualitative IHC assay using rabbit monoclonal anti-PD-L1 clone SP142 [5, 17]. PD-L1 protein on tumor-infiltrating immune cells was assessed in each TMA slide stained with OptiView DAB IHC Detection Kit and OptiView Amplification Kit on a VENTANA BenchMark ULTRA instrument. The scoring of PD-L1 positivity followed the guidelines and where pathologist (YJC) confirmed the tumor-infiltrating immune cells which were morphologically consistent with lymphocytes, macrophages, dendritic cells, and granulocytes by H&E staining. Then, PD-L1 expression on tumor-infiltrating immune cells was quantified in intra-tumoral and peri-tumoral stromal area. Areas with neutrophilic debris and necrotic debris was excluded from scoring. Presence of discernible PD-L1 staining of any intensity covering ≥ 1% of tumor area was considered as PD-L1 positive [5, 9]. Benign tonsil tissue was used as a positive (lymphocytes and macrophages in germinal center, reticulated crypt epithelium) and negative (inter-follicular lesion and overlying squamous epithelium) controls. PD-L1 expression values for each subject are presented as Supplementary Table S1A. The PD-L1 SP142 assay was performed blindly with no clinical information being given to the examiner.

The TIL counts were measured as previously described [13, 18, 19]. TILs were scored according to the standardized methodology proposed by the international TIL Working Group [20]. All mononuclear cells, including lymphocytes and plasma cells, but not polymorphonuclear leukocytes, were counted. The area outside the tumor border, around the intraductal component, and normal lobules were excluded. For each case, the TIL counts were reported as a percentage. CD8 percentage among immune cells was obtained using an anti-CD8 rabbit monoclonal antibody (SP57, Ventana).

### RNA extraction, Affymetrix microarray, and data analysis

Total RNA was isolated using Qiagen RNeasy FFPE kit (Qiagen, Hilden, Germany) according to the manufacturers' instructions. RNA samples extracted from FFPE tissues were analyzed in terms of RNA concentration and purity using NanoDrop ND-1000 Spectrophotometer (NanoDrop Technologies, Wilmington, DE, USA) [21]. Briefly, double-stranded DNA was synthesized using One-cycle cDNA Synthesis Kit (Affymetrix) followed by purification with GeneChip Sample Cleanup Module (Affymetrix). The double-stranded DNA was used as template for the in vitro transcription using Gene Chip IVT PLUS Reagent Kit (Affymetrix), which yields biotinylated cRNA. The purified biotin-labeled target cRNA was then segmented into short sequences. The HG-U133_Plus_2 microarrays (Affymetrix) were directly loaded with 200 µl of hybridization cocktail solution and then placed in Genechip Hybridization Oven 640 (Affymetrix) rotating at 60 rpm at 45 °C for 16 h. After hybridization, the arrays were washed on Genechip Fluidics Station 400 (Affymetrix) and scanned using Genechip. The microarray data were visually inspected for physical damage and background noise. Datasets of all probes were normalized using 100 housekeeping genes in mask files of U-133_Plus_2.0 to a means intensity of 2000 before further date processing.

The gene expression data were normalized by the quantile method, log2 transformed, and median centered across genes and samples. A hierarchical clustering analysis was carried out using Cluster 3.0 [22] and Java Tree View Software. Significance analysis of microarrays with false-discovery rate of 5% was used to identify SP142 PD-L1 related genes and gene signatures [23]. We next applied a collection of 633 known gene signatures, representing multiple biological pathways and cell types, to the median centered and standardized gene expression matrix [24].

Gene expression signatures were calculated as the median expression of all the genes in the signature as published [25]. Univariable and multivariable analyses were performed using standardized signature scores in R-studio using the Cox regression hazard model. Heat-map and PCA plot were used in analysis and DAVID (The Database for Annotation, Visualization and Integrated Discovery) tool was used in functional analysis [26, 27].

### TNBCtype and PAM50 assay

Our RNA dataset was uploaded in the website (http://cbc.mc.vanderbilt.edu/tnbc/) [28], and the TNBCtypes were obtained for each subjects [29]. For PAM50 intrinsic subtypes, we first applied a new HER2/ER subgroup-specific gene normalization method [30], followed by the PAM50 predictor [31]. For each sample, we calculated the correlation coefficient to the PAM50 centroids (Basal-like, HER2-Enriched, Luminal A, Luminal B and Normal-like signatures, respectively) [32].

### External gene expression data analysis

TCGA RNA-seq data were collected as previously described [12, 33]. Samples were limited to TNBC tumors defined by IHC examination. TMB, total neoantigen count, and lymphocyte-infiltration signature score for each subject in the TCGA cohort were determined from a previous publication [34]. RNA-seq data for NCT01560663 is published [35]. Microarray and variant data for the METABRIC cohort [36], was obtained from cBioPortal. RNA-seq data from SCAN-B was downloaded from GEO: GSE96058 [37]. For this study, non-TNBC samples in METABRIC and SCAN-B were excluded.

In urothelial carcinoma, raw sequencing data required for RNA-seq analyses have been deposited to the European Genome-Phenome Archive under accession number EGAS00001002556 [38, 39]. In melanoma, the RNA-seq data are deposited in the European Nucleotide Archive (ENA) [40], under accession number PRJEB23709. For RNA-seq datasets, genes with no reads across any of the samples were removed. Raw RNA-seq data were aligned to reference genome hg38 and quantified using a STAR-Salmon pipeline as previously described [41]. Salmon gene-level counts were upper quartile normalized. Genes with an average expression less than 10 were filtered from the dataset.

### Statistical analyses

The distributions of non-parametric variables were compared using the Mann–Whitney U test. Survival curves were constructed using the Kaplan–Meier product limit method and compared between subtypes with the log-rank test using open-to-public datasets for which DFS, RFS, PFS, and OS results are available. In the training set, the cutoff point of the SP142 gene signature score was obtained using the time-dependent ROC curve. In the cohort of SCAN-B, interaction tests were used to explore differential effects between dichotomized PD-L1-signature score status and chemotherapy treatment in relation to OS. Cox proportional hazard regression model adjusted for available prognostic clinical covariates such as stage was performed to calculate hazard ratios using 95% confidence intervals. Variables showing a statistical significant difference in the univariate analysis were entered in the multivariable analysis. Survival analyses were performed using the R package survival. All statistical analyses were performed using the R software (https://www.r-projet.org; version 3.6.1) and the GraphPad Prism. A *P*-value < 0.05 was considered statistically significant.

### Data and code availability

The published article includes all datasets and code generated in this study. The dataset of 149 TNBC patients generated during this study are available at GEO Datasets: GSE135565 and GSE157284. Newly generated dataset for the current study are available at the NCBI GEO database under accession numbers GSE157284 with the following secure token: qjexicmuldkxjmr. To access the dataset, the editors or reviewers should access the GEO website (https://www.ncbi.nlm.nih.gov/geo/) and insert these secure tokens.

## Results

### Clinical and immunological characteristics of SP142 PD-L1-positive TNBC

**Fig. 1:**
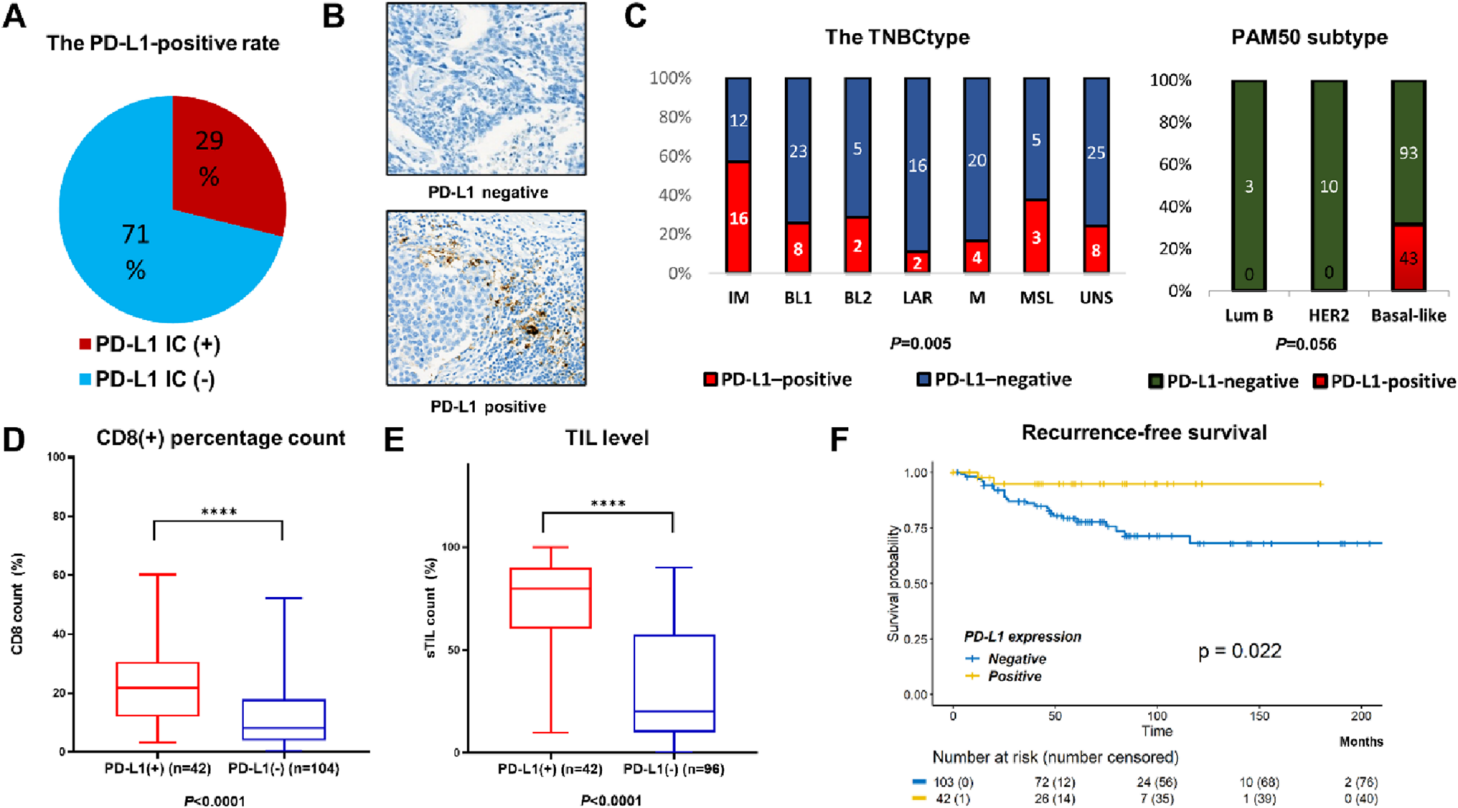
Clinical and pathological characteristics of SP142 PD-L1-positive TNBC. **a** The PD-L1 positive rate according to SP142 expression was 28.9% (43 of 149). **b** Representative images for PD-L1-positive and -negative tumors in high-power fields (× 400 magnification). **c** Left: SP142 PD-L1 percent positivity partitioned by TNBCtype (immunomodulatory [IM], basal-like 1 [BL1], basal-like 2 [BL2], luminal-androgen receptor [LAR], mesenchymal [M], mesenchymal stem-like subtypes [MSL], and unspecified [UNS]). The rate of PD-L1-positive tumors was significantly higher in the IM subtype; the IM subtype had the highest rate of PD-L1 positivity (57.1%), whereas the other subtypes had PD-L1-positivity rates of 10–30% (Chi-square test). Right: PD-L1-positivity rate was not significantly different according to the PAM50 subtype (Chi-square test). **d** PD-L1-positive TNBC had significantly higher mean CD8(+) percentage count (Mann–Whitney U test). **e** PD-L1-positive TNBC had higher mean of TIL counts compared to PD-L1-negative TNBC (Mann–Whitney U test). **f** Recurrence-free survival was significantly prolonged in PD-L1-positve TNBC than in PD-L1-negative TNBC (log-rank test)

To begin to explore the biology and clinical associations of PD-L1 protein expression in TNBC, we collected a set of 149 tumors from which we created TMAs and performed DNA microarray-based gene expression profiling using Affymetrix microarrays. Baseline characteristics of the study population is summarized in Supplementary Table S1B-C. All tumors were treatment-naïve. SP142 PD-L1 expression was successfully determined using these TMAs, and showing a rate of PD-L1+ tumors of 28.9% (43/149; Fig. 1a, Representative images in Fig. 1b).

From the DNA microarray gene expression data, we identified the “TNBC-Type” molecular classification [29] for each subject (Fig. 1c, *left*). When we compared “TNBC-Types” according to PD-L1-positivity, the immunomodulatory subtype had the highest rate of PD-L1 positivity (57.1%), whereas the other subtypes had PD-L1-positivity rates of 10 to 30%. PAM50 subtypes were also evaluated in relation to PD-L1 expression and although this comparison was not significant, all PD-L1+ subjects had basal-like tumors (Fig. 1c, *right*). Next, we noted that PD-L1+ TNBC had significantly higher mean CD8+ cell percentage than PD-L1-negative TNBC (Fig. 1d). In addition, PD-L1+ TNBC had higher average of TIL counts compared to PD-L1-negative TNBC (Fig. 1e).

In survival analyses, we only included non-metastatic TNBCs and excluded one case with missing survival data, leaving 145 subjects available; most of these patients received adjuvant chemotherapy (95%), but none of them were treated with anti-PD-1/PD-L1 inhibitors. The recurrence-free survival (RFS) was significantly prolonged in TNBC patients with PD-L1+ compared to those without PD-L1 (Fig. 1f). In univariable analysis, stage and PD-L1 status were significant prognostic factors (Supplementary Table S1D). In multivariable analysis, PD-L1+ subjects trended to have a better RFS compared to PD-L1-negative (Supplementary Table S1D).

### Gene signature for SP142 PD-L1(+) TNBC

**Fig. 2:**
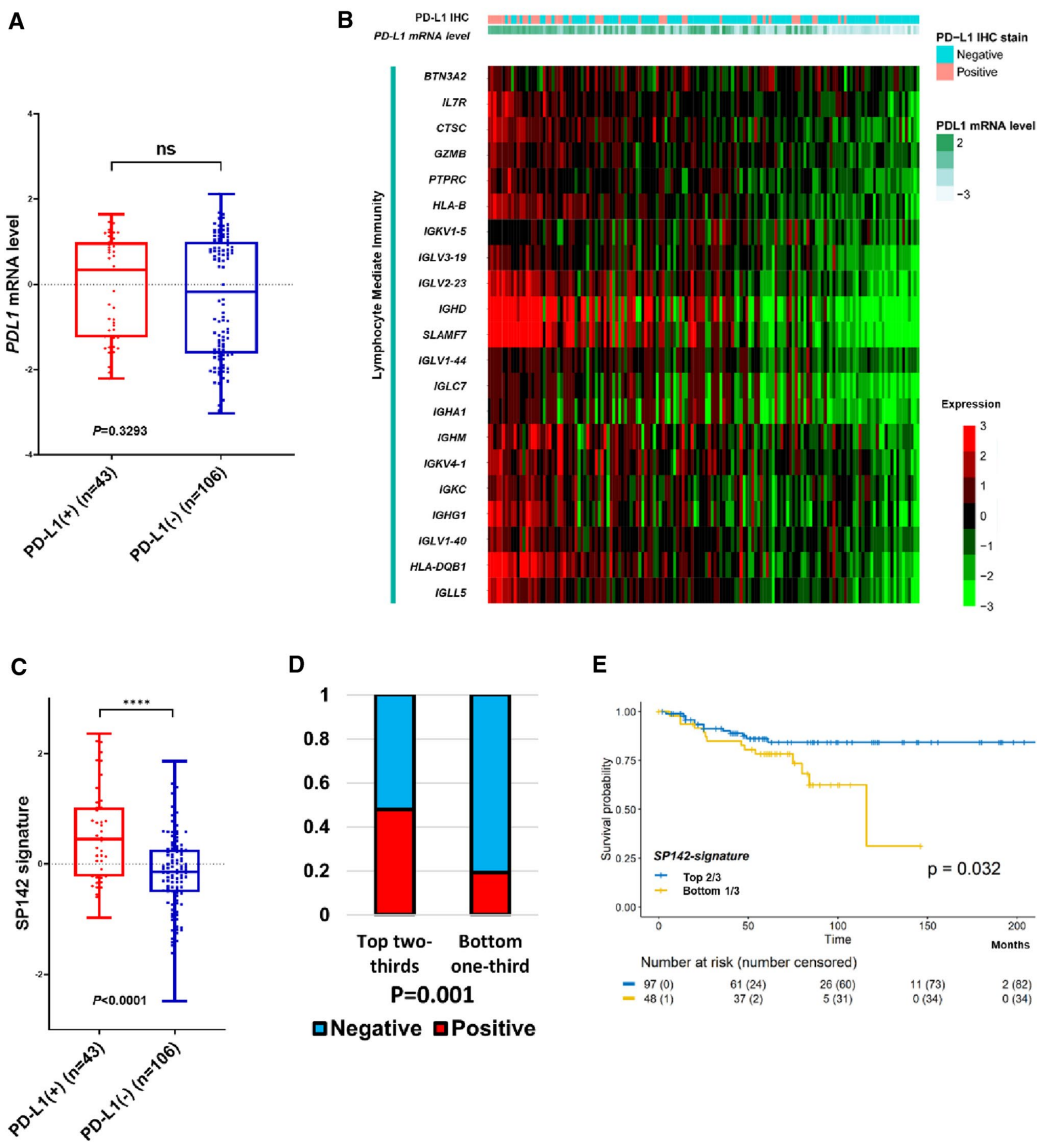
An RNA expression-based SP142 gene signature. **a** PD-L1 mRNA expression in breast tumor samples partitioned by SP142 PD-L1 status (Mann–Whitney U test). **b** A heat-map clustering breast tumors with 21 genes of the “Lymphocyte Mediated Immunity” signature. The rows indicate individual genes, and the columns indicate each sample. PD-L1 IHC status and relative PD-L1 mRNA expression are denoted for each sample above. **c** SP142 signature was significantly higher in PD-L1-positive tumors (Mann–Whitney U test). **d** The proportion of PD-L1 positive patients in the top two-thirds SP142 signature group is significantly higher than in the bottom one-third group (Chi-square test). **e** The top two-thirds SP142 signature group had a superior recurrence-free survival compared to the bottom one-third SP142 signature group (log-rank test)

To begin to explore the possible associations of gene expression patterns with PD-L1 protein expression, we first noted that mean *PD-L1* mRNA expression was not significantly different by SP142 PD-L1 protein assay status (Fig. 2a). Thus, we conducted supervised analyses according to PD-L1 protein positivity to identify a gene expression pattern associated with SP142 PD-L1 in TNBC. A two-class significance analysis of microarrays (SAM) [23] supervised analysis identified 94 highly expressed genes in PD-L1+ TNBC (listed as Supplementary Table S2, expression heatmap as Supplementary Fig. S1). A majority of this gene cluster, which is henceforth referred to as SP142 signature, consisted of immune-related genes including immunoglobulins, major histocompatibility genes, and chemokines. To better understand the biological functions underlying the SP142 signature, we explored the gene ontology (GO) associated with this 94 gene set. This analysis strongly implicated a response of the immune system, largely mediated through adaptive immunity (Supplementary Table S2). The extent of lymphocyte activity in PD-L1+ patients is well illustrated by a heatmap showing expression of the 21 genes associated with the GO term “lymphocyte mediated immunity (GO:0002449)” and *PD-L1* mRNA levels (Fig. 2b).

We next calculated a SP142 signature score in our 149 subjects and found that, as expected, the mean score was significantly higher in PD-L1+ TNBC than in PD-L1-negative (Fig. 2c). Next, we tested the prognostic value of SP142 signature. The continuous SP142 signature score was prognostic for RFS (HR 0.535, 95% CI 0.328–0.872) on this set of 145 non-metastatic TNBC patients. To utilize the SP142 signature as a prognostic categorical value, the time-dependent receiving operating characteristic (ROC) curve in relation to RFS was used to identify the optimal cutoff (Supplementary Fig. S2A). As a result, the top two-thirds were selected for the better RFS group versus the bottom third. Biologically, cases with top two-thirds of SP142 signature score had higher CD8 and TIL counts than those with bottom one-third (Supplementary Fig. S2B-C). Based on these findings, we classified our patients into two groups by SP142 signature; top two-thirds and bottom one-third. This assignment rate also reflects the findings of a recent clinical trial showing the PD-L1-positive rate as 56% in early TNBC [11].

The top two-thirds group had a significantly higher rate of PD-L1+ tumors (Fig. 2d), as was expected. When survival analyses were performed between these groups, we found that patients in the top two-thirds had a superior RFS when compared to patients in the lowest third for SP142 signature (Fig. 2e). Multivariable analyses including stage further supported the independent prognostic value of SP142 signature (Table 1). In contrast, continuous or categorical values of PD-L1 mRNA levels alone were not associated with RFS in the same set (Supplementary Fig. S3), indicating that SP142 signature was a better prognostic factor than PD-L1 protein expression in infiltrating immune cells (which only trended in multivariable analyses), or *PD-L1* mRNA levels on this set.

**Table 1:** The Cox regression hazard model with SP142 signature and stage

	Recurrence-free survival						
	HR	95% CI	*P*		HR	95% CI	*P*
SP142 signature			0.026				
Continuous							
	0.546	0.321–0.930		Bottom one	1		0.049
				Top two	0.463	0.215–0.996	
Stage							
I	1			I	1		
II	1.647	0.585–4.636	0.345	II	1.638	0.579–4.636	0.352
III	7.639	2.551–22.876	< 0.0001	III	8.081	2.696–24.223	< 0.0001

### Clinical value of SP142 signature in early TNBC

**Fig. 3:**
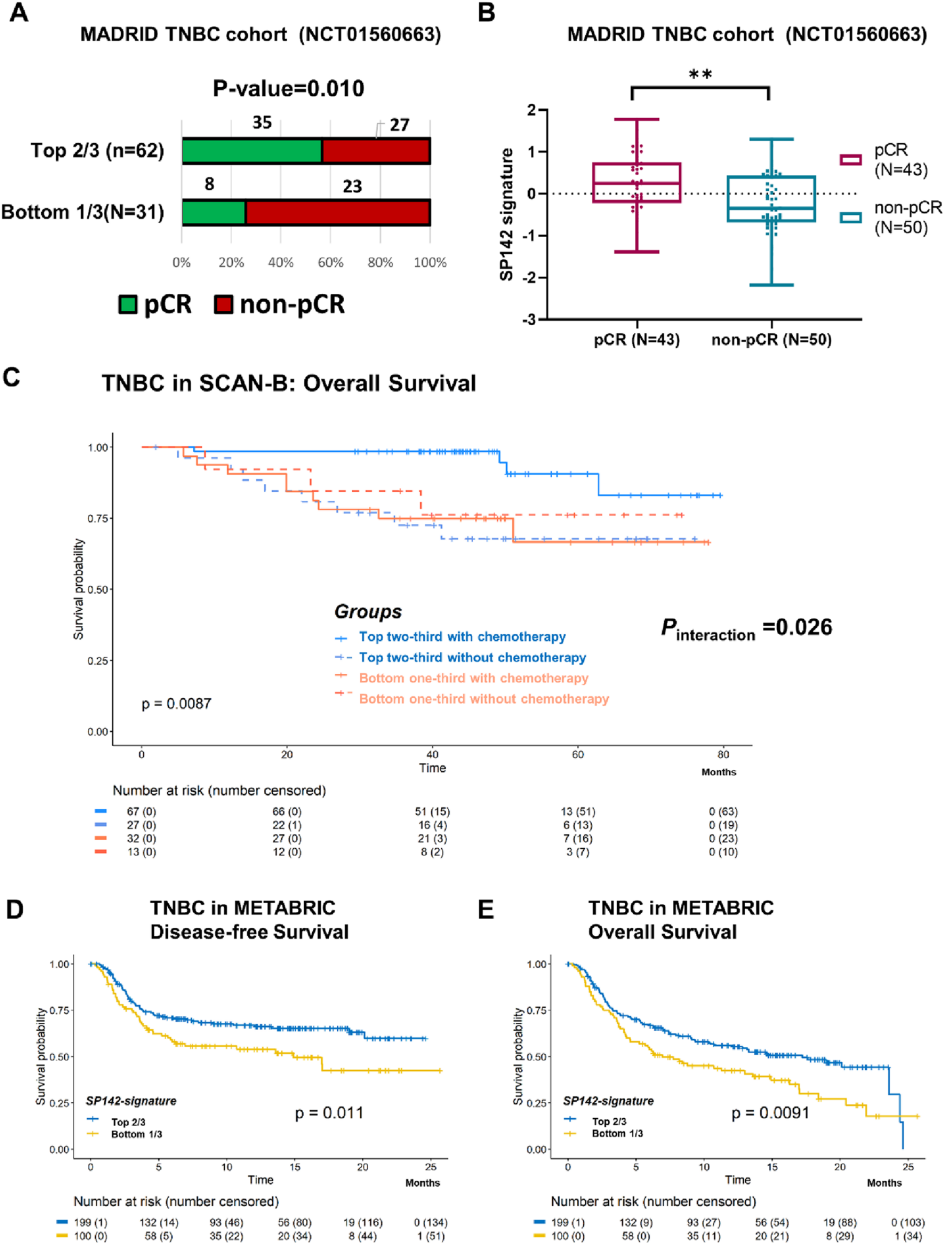
Clinical value of SP142 signature in TNBC. **a** In the Madrid cohort, (NCT01560663), pathologic complete response (pCR) rate was higher in the SP142 signature top two-thirds group (Chi-square test). **b** SP142 signature score was significantly higher in the pCR group than in non-pCR group (unpaired *T*-test). **c** Overall Survival (OS) in TNBC from SCAN-B, with patients classified by SP142 signature and chemotherapy treatment status., Patients in the SP142 signature top two-thirds group that received chemotherapy had better OS (the log-rank test). The interaction test between chemotherapy treatment and dichotomized SP142 signature status was significant (*P*_interaction_ = 0.026). **d** Disease-free, and **e** overall survival in TNBC patients of the METABRIC cohort. The SP142 signature top two-thirds group had significantly better prognosis (log-rank test)

We next addressed the possible clinical value of the SP142 signature in early TNBC with respect to pCR and survival outcomes. First, we used published data from a TNBC cohort of 93 patients undergoing preoperative docetaxel/carboplatin chemotherapy (NCT01560663) [42]. The SP142 signature-high subset of patients had a higher pCR rate (Fig. 3a), and when analyzed as a continuous score, patients with a pCR had a significantly higher SP142 scores (Fig. 3b). These findings suggest that a high SP142 signature might be associated with a higher likelihood of response to neoadjuvant chemotherapy in TNBC.

To test a relationship between SP142 signature and adjuvant chemotherapy responsiveness, we used RNA sequencing data in TNBC samples from the Sweden Cancerome Analysis Network-Breast Initiative (SCAN-B) cohort [37]. First, patients with higher SP142 signature had significantly better OS (Supplementary Fig. S4A), which was even more significant when the patient set was limited to TNBC patients receiving chemotherapy (Supplementary Fig. S4B). When we classified the patients into four groups by SP142 signature and chemotherapy treatment, chemotherapy improved OS in the SP142 top two-thirds group whereas it did not for those in the SP142 signature bottom one-third group (Fig. 3c). The interaction test between chemotherapy and dichotomized SP142 signature status was significant (*P*_interaction_ = 0.026), however, we acknowledge that this is not a randomized trial, and thus further testing is warranted.

Lastly, we evaluated the prognostic value of SP142 signature in another non-metastatic TNBC cohort from Molecular Taxonomy of Breast Cancer International Consortium (the METABRIC cohort) [37]. In this patient subset, both disease-free survival (DFS) and OS were superior in the SP142 signature top two-thirds group than in the lowest third group (Fig. 3d, e).

### Immunologic characterization for SP142 signature using TCGA

**Fig. 4:**
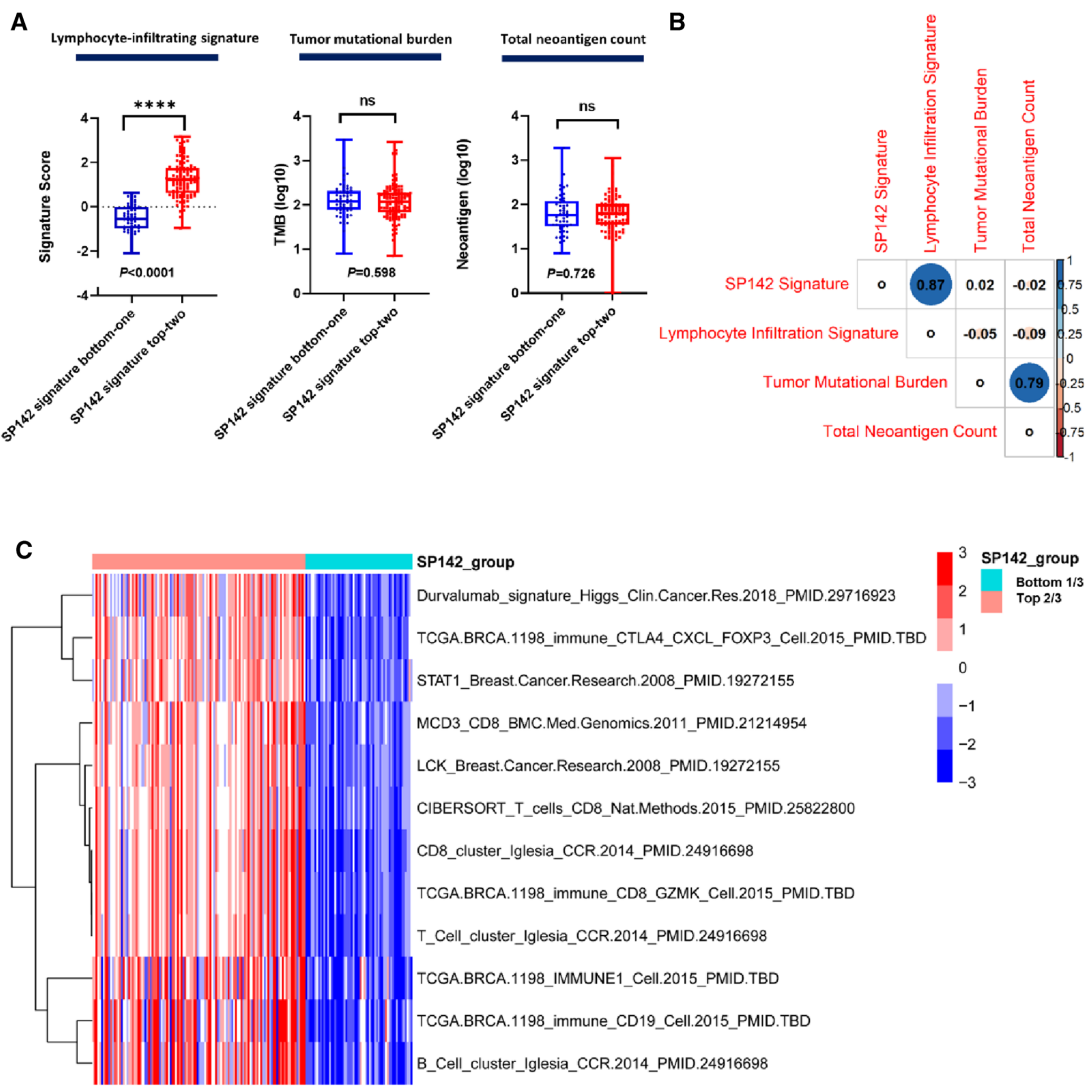
Immunologic characterization for SP142 signature in the TNBC of TCGA. **a** Left: The lymphocyte-infiltrating signature score was significantly higher in the top two-thirds SP142 signature group than in the bottom one-third group (Mann–Whitney U test). Tumor mutational burden (*middle*) and total neoantigen count (*right*) did not significantly differ between the top two-thirds and bottom one-third SP142 signature groups (Mann–Whitney U test). **b** The SP142 signature score shows strong correlation with the lymphocyte-infiltration score, but not with correlated with total mutation burden (TMB) or total neoantigen count. TMB and total neoantigen were also correlated (Pearson’s *R*-test). The numbers in the graph indicate Spearman rank correlation coefficients. **c** Heat-map displaying immune-related gene signatures with > fourfold increase between SP142 signature top two-thirds and bottom one-third groups (SAM analysis)

Next, we applied the SP142 signature to TNBC from TCGA. We divided tumors into two groups as the top two-thirds and bottom third using SP142 signature. We compared lymphocyte infiltration-signature score [43] between these two group, revealing that lymphocyte-infiltration signature score (as determined by the TCGA immune Pan-Cancer analysis) was significantly increased in SP142 signature-high patients, consistent with the increased TIL counts that were noted in SP142 PD-L1+ TNBC (Fig. 4a). We investigated whether differences in somatic mutation or neoantigen burden could explain the differences between these patient subsets, however, neither of these measures differed between groups (Fig. 4a) and strong correlations were only found between SP142 signature and lymphocyte-infiltrating score or TMB and total neoantigen count (Fig. 4b).

To further validate the biological implications of the SP142 signature, we examined the possible association of this signature when compared to 633 previously published gene signatures [24, 44]. Using SAM analysis provided additional evidence of the strong association between the SP142 gene signature and an active immune response where we found 12 immune pathway-enriched signatures with at least fourfold increase in the SP142 signature top two-thirds samples (Fig. 4c and Supplementary Table S3).

### SP142 signature in other malignancies treated with ICIs

**Fig. 5:**
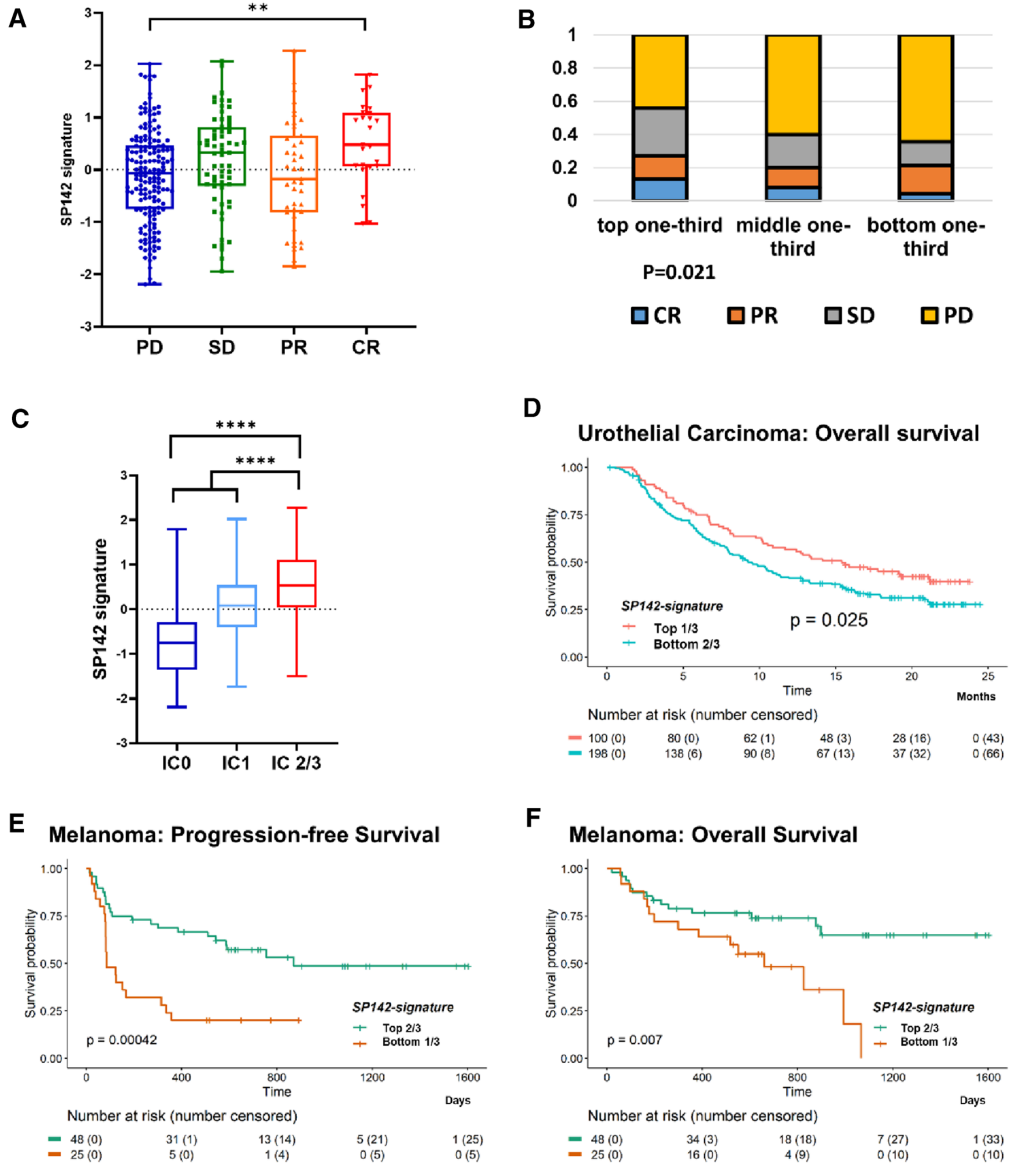
SP142 signature in other malignancies treated with immune check-point inhibitors (ICIs). **a**–**d** In metastatic urothelial carcinoma patients treated with atezolizumab, **a** SP142 signature score in patients partitioned by treatment response (*PD* progressive disease, *SD* stable disease, *PR* partial response, *CR* = complete response). The complete response (CR) group showed the highest mean SP142 signature score (one-way ANOVA test). **b** Response rates in patients partitioned by SP142 signature scores tertiles. The CR rate was higher, while PD was lower in top one-third than in either middle or bottom one-third groups (the Chi-square test). **c** SP142 signature score in the subsets of patients by VENTANA SP142 assay classification (IC0 = PDL1 expressing immune cells < 1%, IC1 (≥ 1% and < 5%), IC2/3 (≥ 5%)). SP142 score showed an increasing pattern according to the IC groups (one-way ANOVA test, *P* < 0.001; unpaired *T*-test between IC0 and IC1, *P* < 0.0001; unpaired *T*-test between IC1 and IC2/3, *P* < 0.0001). **d** Overall survival (OS) of patients grouped with the top one-third SP142 signature score versus the bottom two-thirds (log-rank test). **e**, **f** In melanoma patients treated with immune-check point inhibitors, progression-free (**e**), and overall survival (**f**) are significantly better for the group of patients in the top two-thirds of SP142 signature score relative to the lowest one-third group (log-rank test)

SP142 is an assay used to select cancer patients to receive immune checkpoint inhibitors, and given that no publically available TNBC ICI-treated data set exists, we turned to other tumor types to evaluate the relationship between the SP142 signature and ICI responsiveness. First, we tested our gene signature on a metastatic urothelial carcinoma cohort treated with atezolizumab [38, 39]. Comparison of mean SP142 signature by response to atezolizumab found that patients that experienced a complete response (CR) showed the highest score (Fig. 5a). Also, in the group with top one-third SP142 signature, the CR rate was higher, and the rate of progressive disease (PD) was lower, than in either the middle or bottom one-third groups (Fig. 5b).

In this cohort, PD-L1 expression on immune cells (IC) was assessed by the SP142 assay, which is identical to the assay used in our 149 TNBCs, though the urothelial carcinoma cohort was score as IC0 (< 1%), IC1 (≥ 1% and < 5%), or IC2/3 (≥ 5%). The SP142 signature score showed an increasing pattern according to the IC groups (Fig. 5c). In survival analysis, a continuous SP142 signature was prognostic for OS (HR 0.815, 95% CI 0.699–0.951), and furthermore, the group with top one-third SP142 signature score showed a better OS than the group with bottom two-thirds (Fig. 5d).

Lastly, we evaluated our SP142 signature in a melanoma cohort treated with ICIs [40, 45]. This cohort consisted of 73 patients treated with either anti-PD-1 therapy or combination blockade of PD-1 and CTLA-4 (Supplementary Table S4A). A continuous SP142 signature was prognostic for both PFS and OS (Supplementary Table S4B). Further survival analyses showed that the top two-thirds patients had a superior PFS and OS compared to bottom one-third patients (Fig. 5e, f).

## Discussion

In this study, we investigated the clinical and genomic characteristics of SP142 PD-L1+ TNBC relative to SP142 PD-L1-negative TNBC. Our findings demonstrate that SP142 PD-L1+ tumors have more immunogenic traits, including high TILs by H&E, higher CD8 counts by IHC, enrichment of immune TNBC genomic subtype and showed elevated immune gene signatures at the gene expression level. Since several lines of evidence suggested that the SP142 assay detects more immune cells but fewer tumor cells compared to other PD-L1 assays [46, 47], and thus it is expected that SP142-positive TNBCs would be enriched with TILs, CD8+ cells, and other immune features, and they were. Intriguingly, *PD-L1* mRNA expression level did not differ according to SP142 PD-L1 status. Despite a few earlier studies showing that PD-L1 mRNA was correlated with PD-L1 expression by SP142 assay in various malignancies [48–50], the recent study conducted in TNBC consistently showed that the levels of *PD-L1* mRNA were not statistically significantly different between the SP142-based categorical value (IC ≥ 1 versus IC < 1) [51]. In addition, another study reported that the protein expressional level of PD-L1 in tumor and immune cells using SP142 assay was significant lower than in other assays [52]. Association between SP142-detected PD-L1 expression on immune cells and *PD-L1* mRNA level should be further evaluated and may vary according to tumor/anatomic site as well.

Further genomic analyses elucidated that multiple immune-related genes were enriched in SP142 PD-L1-positive tumors. By generating a SP142 signature, we were able to classify TNBC tumors from additional datasets that had not had PD-L1 IHC classification, thus showing that our gene signature classification provided meaningful prognostic information on multiple TNBC cohorts. In addition, we showed that on the 149 patient TNBC set, the PD-L1 signature was a better prognostic factor than SP142 protein expression, or PD-L1 mRNA expression, noting that this is a training set exercise for the SP142 signature.

In addition, the SP142 signature was biologically validated using RNAseq data of TCGA-TNBC. This genomic study showed that the SP142 signature was correlated with lymphocyte-infiltration signature but not associated with TMB or neoantigen count, indicating that immunogenicity of TNBC arises mainly from lymphocytes-infiltrated microenvironment. In addition, TNBCs with high SP142 signature exhibit increased expression of many other immune gene signatures compared to those with low-SP142 signature. Our findings are consistent with a prior study showing that the rate of high TMB tumors in TNBC is less than 10%, while the frequency of high T cell-inflamed TNBC samples is about 50% [2]. Collectively, our data indicates that increased level of lymphocyte infiltration is a major factor contributing to SP142 PD-L1 positivity in TNBC.

A hypothesis based upon the SP142 signature is the ability to distinguish TNBC patients that are likely to respond to combined ICIs and chemotherapy, and to do so objectively and in a quantitative fashion. Because a prior study suggested that a T cell-inflamed gene signature might be potential biomarker responding to pembrolizumab in TNBC [2], investigating the SP142 signature as a predictive biomarker for PD-1/PD-L1 inhibitors is a promising future study.

This line of investigation is further strengthened by our evaluation of the SP142 signature in urothelial carcinoma, and melanoma cohorts, in which the patients were treated with ICIs. In metastatic urothelial carcinoma, the SP142 signature was associated with CR to atezolizumab and correlated with the level of PD-L1 expression on immune cells. In both cohorts, continuous or categorical SP142 signature was associated with a better survival outcome; however, we note the use of the top one-third of SP142 scores to differentiate survival in urothelial carcinoma, as opposed to the top two-thirds used in TNBC and melanoma. This is likely attributable to differences in biology among various malignancies. Nonetheless, these findings suggest that the SP142 signature may be a valuable biomarker for ICI response in TNBC as well as other cancers, however, tumor type specific cutpoints may be required for optimal implementation.

Clinically, we recognized that PD-L1-positive TNBCs tended to have a better RFS than PD-L1-negative TNBC. The positive result showing PD-L1-positivity detected by standardized SP142 assay as a strong predictor for pCR in early TNBC [11] supports the notion that PD-L1 positivity on intra-tumoral immune cells is a favorable prognostic factor. It is known that an increment of pCR often correlates with better survival outcome in TNBC [53], suggesting that patients with SP142 PD-L1 expression might have a better outcome than those without expression; further analyses are needed to confirm this hypothesis.

These results demonstrate potential clinical value of the SP142 signature for pCR and survival in TNBCs, with the important implication that a high SP142 signature is associated with a higher likelihood of response to chemotherapy based on the neoadjuvant and adjuvant cohorts tested (Fig. 3a–d). Taken together, our survival analyses suggest consistently that the subjects identified by SP142 PD-L1 assay, or SP142 gene signature, have a more favorable prognosis among TNBC patients. Consistent with our results, a body of evidence already showed that high TIL-tumors have better outcome in TNBC [54–56].

A major caveat of our study is that we evaluated PD-L1 expression using TMA. Since the SP142 system evaluates PD-L1 expression on immune cells, contiguous peritumoral stroma could be investigated using the whole slide, but not on TMAs. Core extraction for TMAs focuses primarily on invasive tumor components with a criteria of high cellularity (> 80%), making it possible that peritumoral stroma might not be fully evaluated in some cases, thereby increasing the potential for false negative cases in our study. This is a potential reason why our PD-L1 positive rate of 29% may be lower than published atezolizumab studies, which showed the rate as 41% in metastatic [5] or 56% in early TNBC [11]. Nevertheless, the PD-L1+ samples identified in this study represent true positive cases in this context, thus our findings likely reflect genuine characteristics of those tumors. Given that the primary goal of this study was to explore the biological and clinical traits of SP142+ TNBC, validating the true clinical utility of SP142 PD-L1 expression or SP142 genomic signature is beyond the scope of this study, but clearly the next step for this signature.

In conclusion, we provide multi-faceted evidence that SP142 PDL1+ TNBC have immuno-genomic features characterized as highly lymphocyte-infiltrated and a relatively favorable survival amid TNBC. In addition, SP142 genomic signature might provide information to identify immunogenic tumors making up as much as two-thirds of TNBC. These results enhance our understanding about immunogenicity of TNBC and lay the groundwork for further studies integrating IHC and gene expression immune information for selecting patients to receive PD-1/PD-L1 target therapies.

## Supplementary Information

Below is the link to the electronic supplementary material.

Supplementary file1 (PDF 444 kb)

Supplementary file2 (PDF 315 kb)

Supplementary file3 (PDF 331 kb)

Supplementary file4 (PDF 355 kb)

Supplementary file5 (PDF 402 kb)

Supplementary file6 (XLSX 52 kb)

Supplementary file7 (PDF 115 kb)

Supplementary file8 (PDF 209 kb)

## Data Availability

Further information and requests for materials may be directed to the corresponding author Charles M Perou. (cperou@med.unc.edu).

## Code availability

The published article includes all datasets and code generated in this study. The dataset of 149 TNBC patients generated during this study are available at GEO Datasets: GSE135565 and GSE157284. Newly generated dataset for the current study are available at the NCBI GEO database under accession numbers GSE157284 with the following secure token: qjexicmuldkxjmr. To access the dataset, the editors or reviewers should access the GEO website (https://www.ncbi.nlm.nih.gov/geo/) and insert these secure tokens.
