# Supplementary material for: Clinical and genomic assessment of PD-L1 SP142 expression in triple-negative breast cancer
Source: Breast Cancer Res Treat. 2021 Mar 26;188(1):165–78. doi: 10.1007/s10549-021-06193-9 (PMC8233296; doi:10.1007/s10549-021-06193-9)

# Supplementary Figure S1. 94 genes of SP142 signature

A. The list of 94 gene in SP142 signature

B. Heat-map by 94 genes of SP142 signature, PD-L1 IHC stain, and PD-L1 mRNA

**A**

| Gene_symbol | Fold.Change | q.value |
|---|---|---|
| SEPTIN6 | 1.456031796 | 0 |
| AIM2 | 1.73486923 | 0 |
| APOL3 | 1.554393026 | 0 |
| BTN3A2 | 1.465665444 | 0 |
| C4orf7 | 2.921203032 | 0 |
| CARD16 | 1.579350303 | 0 |
| CCL8 | 1.870616013 | 0 |
| CD47 | 1.592871186 | 0 |
| CD53 | 1.49544661 | 0 |
| CD84 | 1.531504712 | 0 |
| CDC42SE2 | 1.777040804 | 0 |
| CTSC | 1.736296833 | 0 |
| CXCL10 | 2.168837826 | 0 |
| CXCL11 | 1.584706412 | 0 |
| CXCL13 | 1.650543767 | 0 |
| CXCL9 | 2.334644391 | 0 |
| CYTIP | 1.481208892 | 0 |
| DOCK8 | 1.469104811 | 0 |
| DTX3L | 1.610574583 | 0 |
| FCRL5 | 1.784310759 | 0 |
| FYB | 1.784667974 | 0 |
| GABBR1 | 2.485921132 | 0 |
| GBP1 | 2.176387458 | 0 |
| GBP2 | 1.477973179 | 0 |
| GBP5 | 1.757652962 | 0 |
| GLYR1 | 1.643739527 | 0 |
| GZMB | 1.513681641 | 0 |
| HCLS1 | 1.661828451 | 0 |
| HLA-B | 1.534944565 | 0 |
| HLA-C | 1.539062929 | 0 |
| HLA-DMA | 1.509871627 | 0 |
| HLA-DQA1 | 2.137190584 | 0 |
| HLA-DQB1 | 1.494802449 | 0 |
| HLA-DRA | 1.636967018 | 0 |
| HLA-F | 1.68599939 | 0 |
| IDO1 | 1.815018052 | 0 |
| IFI44 | 1.684373428 | 0 |
| IFI44L | 1.969338975 | 0 |
| IFIH1 | 1.514415288 | 0 |
| IFIT3 | 1.607671977 | 0 |
| IFITM1 | 1.631847914 | 0 |
| IGHA1 | 2.067112875 | 0 |
| IGHD | 1.897592473 | 0 |
| IGHG1 | 2.08372647 | 0 |
| IGHM | 2.216039279 | 0 |
| IGJ | 1.662597127 | 0 |
| IGK@ | 1.980287338 | 0 |
| IGKC | 1.728537543 | 0 |
| IGKV1-5 | 1.665938791 | 0 |
| IGKV4-1 | 1.807790939 | 0 |
| IGL@ | 2.235427528 | 0 |
| IGLC7 | 1.540237468 | 0 |
| IGLJ3 | 1.655147374 | 0 |
| IGLL5 | 1.629262124 | 0 |
| IGLV1-36 | 2.180070062 | 0 |
| IGLV1-40 | 1.637497841 | 0 |
| IGLV1-44 | 1.780611126 | 0 |
| IGLV2-23 | 1.663779997 | 0 |
| IGLV3-19 | 1.65102181 | 0 |
| IKZF3 | 1.768663993 | 0 |
| IL2RG | 1.779536862 | 0 |
| IL32 | 1.591022444 | 0 |
| IL7R | 1.494497123 | 0 |
| KLHDC7B | 1.819850064 | 0 |
| KLHL6 | 1.478670981 | 0 |
| LCP2 | 1.496950843 | 0 |
| LYZ | 2.039777892 | 0 |
| MS4A1 | 1.753765448 | 0 |
| MX1 | 1.526141228 | 0 |
| NLRC5 | 1.67286041 | 0 |
| PLAC8 | 1.578135635 | 0 |
| PLEK | 1.505904951 | 0 |
| POU2AF1 | 1.794345872 | 0 |
| PTPRC | 1.558155897 | 0 |
| RARRES1 | 1.585168446 | 0 |
| RDH10 | 1.70694934 | 0 |
| SAMD9L | 1.703179347 | 0 |
| SAMHD1 | 1.553074527 | 0 |
| SEL1L3 | 1.816060349 | 0 |
| SELL | 1.472481827 | 0 |
| SLAMF7 | 2.039456219 | 0 |
| SLAMF8 | 1.518592622 | 0 |
| SLFN5 | 1.456119654 | 0 |
| SP110 | 1.636067426 | 0 |
| SRGN | 1.481033423 | 0 |
| STAT1 | 2.021491556 | 0 |
| STK17B | 1.573745409 | 0 |
| TAP1 | 1.685149145 | 0 |
| TRAC | 1.442462552 | 0 |
| TRBC1 | 1.637350972 | 0 |
| TRDV3 | 1.611717854 | 0 |
| WARS | 1.538215027 | 0 |
| XAF1 | 1.654487362 | 0 |
| ZBP1 | 1.585187776 | 0 |

**B**

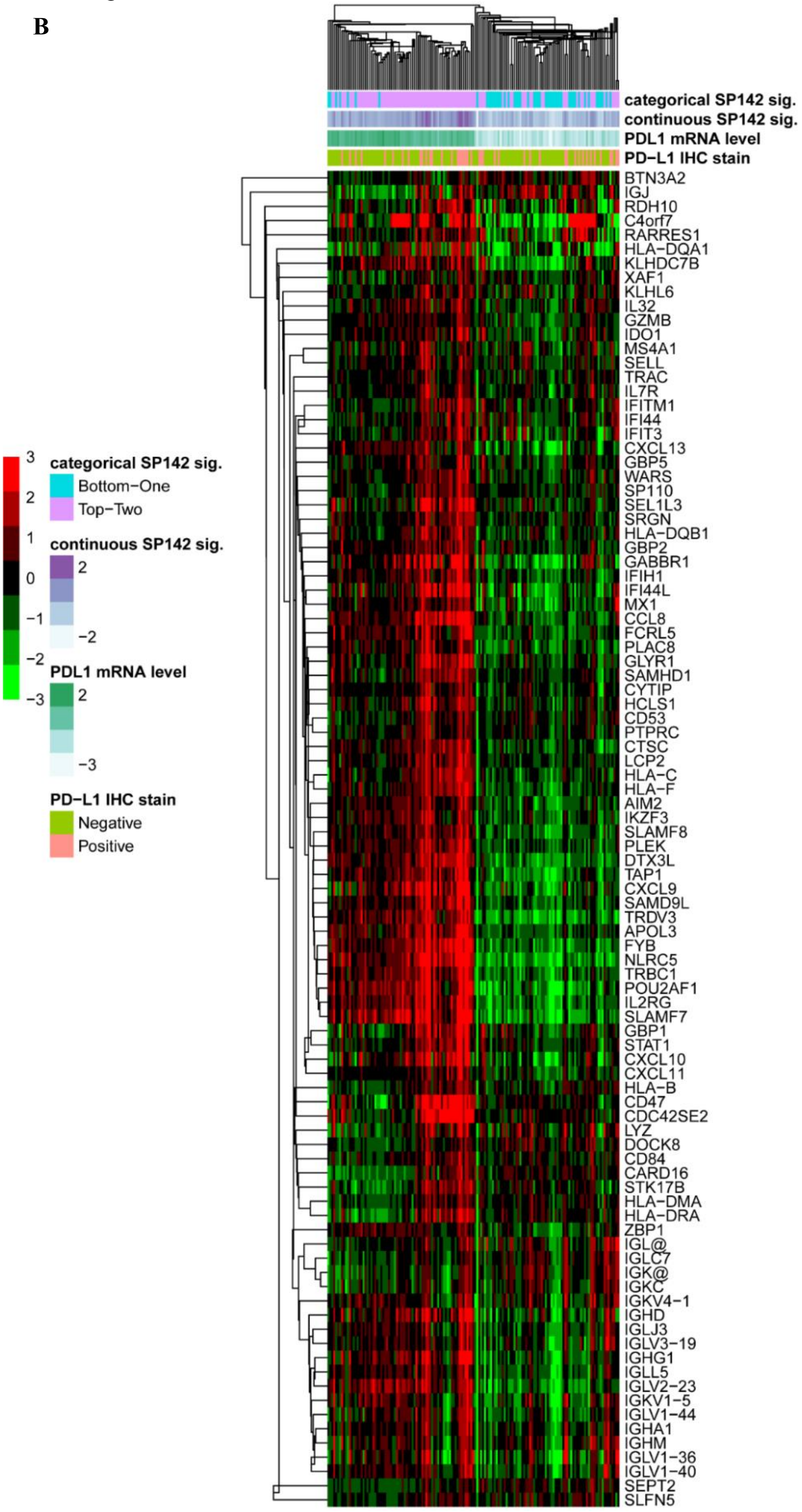

Supplement: Supplementary file 1 — Supplementary file1 (PDF 444 kb) [file 10549_2021_6193_MOESM1_ESM.pdf]
