# Supplementary material for: Clinical and genomic assessment of PD-L1 SP142 expression in triple-negative breast cancer
Source: Breast Cancer Res Treat. 2021 Mar 26;188(1):165–78. doi: 10.1007/s10549-021-06193-9 (PMC8233296; doi:10.1007/s10549-021-06193-9)

Supplementary Figure S2. Identification of the cutoff of SP142 signature for a prognosis

(A) The ROC curve of SP142 signature was obtained in relation to RFS. The AUC was 0.674 (0.568-0.780). Youden's index was -0.402 of median scaled SP142 score. Two-thirds was determined as a cutoff for a prognostic discrimination.

(B) Mean TIL counts were higher in the top or middle one-thirds of SP142 score than in the bottom one-third (one-way ANOVA test, $P<0.001$; unpaired T-test between top and middle one thirds, $P=0.664$; unpaired T-test between middle and bottom one-thirds, $P=0.001$).

(C) Mean TIL counts were higher in the top or middle one-thirds of SP142 score than in the bottom one-third (one-way ANOVA test, $P=0.001$; unpaired T-test between top and middle one thirds, $P=0.4290$; unpaired T-test between middle and bottom one-thirds, $P=0.003$).

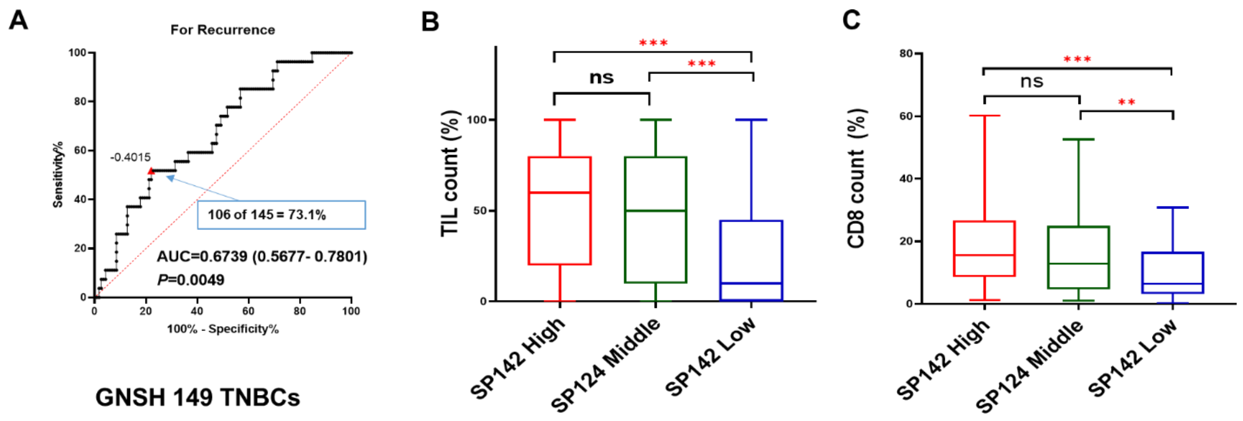

Supplement: Supplementary file 2 — Supplementary file2 (PDF 315 kb) [file 10549_2021_6193_MOESM2_ESM.pdf]
