# Supplementary material for: Clinical and genomic assessment of PD-L1 SP142 expression in triple-negative breast cancer
Source: Breast Cancer Res Treat. 2021 Mar 26;188(1):165–78. doi: 10.1007/s10549-021-06193-9 (PMC8233296; doi:10.1007/s10549-021-06193-9)

**Supplementary Figure S3. Clinical value of single PD-L1 mRNA level in the training set**

In the Cox-regression model, continuous PD-L1 mRNA level was not predictive of the recurrence-free survival in the training set. Additionally, categorical value of PD-L1 mRNA level (top two-thirds vs. bottom one-third) was not significant for a prognostic differentiation by the log-rank test.

| | HR | 95% CI | P-value |
|---|---|---|---|
| **Continuous PD-L1 mRNA** | 0.514 | 0.213-1.241 | 0.139 |

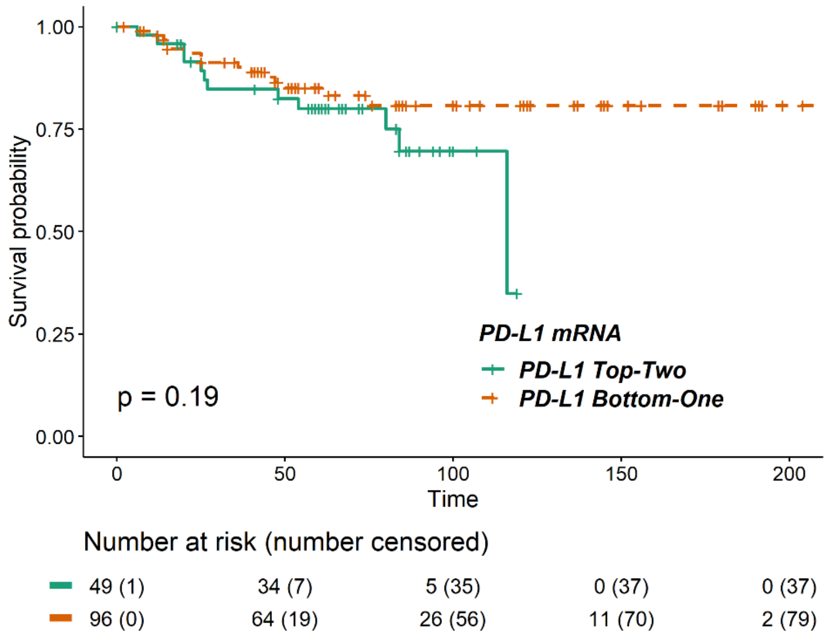

Supplement: Supplementary file 3 — Supplementary file3 (PDF 331 kb) [file 10549_2021_6193_MOESM3_ESM.pdf]
