# Supplementary material for: Clinical and genomic assessment of PD-L1 SP142 expression in triple-negative breast cancer
Source: Breast Cancer Res Treat. 2021 Mar 26;188(1):165–78. doi: 10.1007/s10549-021-06193-9 (PMC8233296; doi:10.1007/s10549-021-06193-9)

Supplementary Figure S4. Clinical value of SP142 signature in TNBC from SCAN-B

(A) Overall survival was prolonged in the top two-thirds of patients (*P*=0.048, the log-rank test).

(B) Among the patients receiving chemotherapy, OS was also superior in the top two-thirds of patients (*P*=0.0035, the log-rank test).

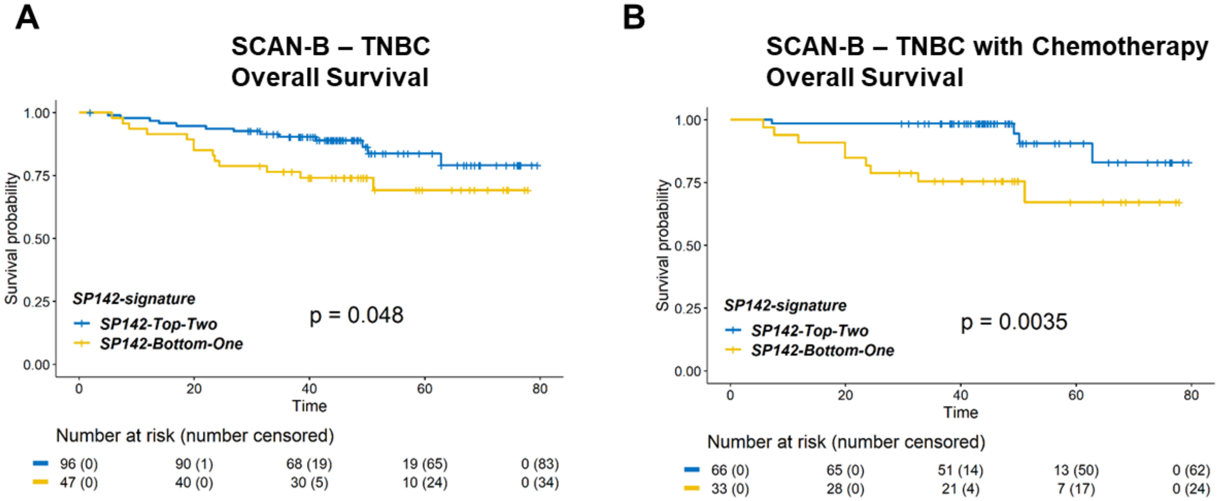

Supplement: Supplementary file 4 — Supplementary file4 (PDF 355 kb) [file 10549_2021_6193_MOESM4_ESM.pdf]
