# Supplementary material for: Clinical and genomic assessment of PD-L1 SP142 expression in triple-negative breast cancer
Source: Breast Cancer Res Treat. 2021 Mar 26;188(1):165–78. doi: 10.1007/s10549-021-06193-9 (PMC8233296; doi:10.1007/s10549-021-06193-9)
Supplement: Supplementary file 5 — Supplementary file5 (PDF 402 kb) [file 10549_2021_6193_MOESM5_ESM.pdf]

Supplementary Table S1. The TNBC cohort with SP142 PD-L1 evaluation (n=149)

A. Distribution of the results of PD-L1 immune cell percentage counts

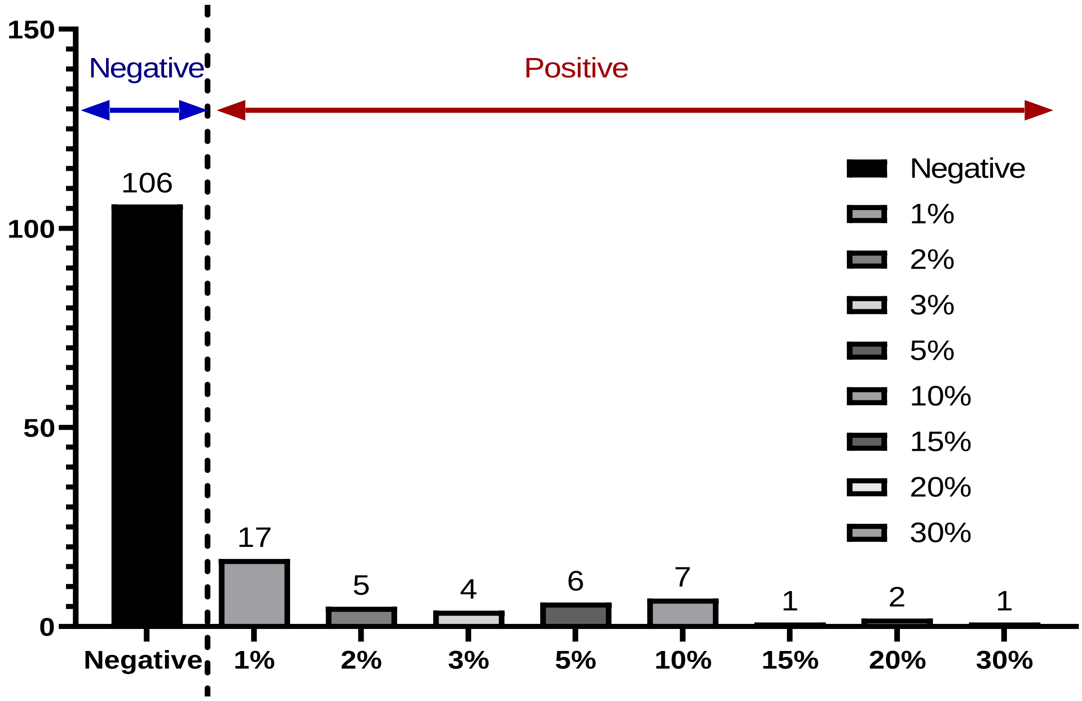

B. Clinical and pathological characteristics

	All (N=149)
AGE	51.9 ± 23.4
Histology	
Invasive ductal carcinoma	115 (77.2%)
Medullary carcinoma	13 (8.7%)
Metaplastic carcinoma	7 (4.7%)
Apocrine carcinoma	6 (4.0%)
Others	8 (5.4%)
T stage	
1	61 (40.9%)
2	76 (51.0%)
3	11 (7.4%)
Missing	1 (0.7%)
N stage	
0	104 (69.8%)
1	30 (20.1%)
2	9 (6.0%)
3	5 (3.4%)
Missing	1 (0.7%)
AJCC stage	
1	54 (36.2%)
2	76 (51.0%)
3	15 (10.1%)
4	3 (2.0%)
Missing	1 (0.7%)
Histologic grade	9 (6.0%)
1	28 (18.8%)
2	107 (71.8%)
3	5 (3.4%)
Missing	
Ki67	36.3 ± 21.4
Chemotherapy	
No	7 (4.7%)
Yes	141 (94.6%)
Missing	1 (0.7%)

C. Type of chemotherapy (n=141)

Anthracycline	77 (54.6%)
Anthracycline + Taxane	45 (31.9%)
Platinum-based regimen	1 (0.7%)
Oral 5-fluorouracil	13 (9.2%)
Cyclophosphamide+Methotrexate+5-fluorouracil	5 (3.6%)

D. The Cox regression hazard model for recurrence-free survival

		Univariable analysis		
		**Hazard ratio**	**95% CI**	**P-value**
**Age**		1.005	0.995-1.014	0.317
**Stage**	I	1		
	II	1.756	0.623-4.949	2.867
	III	8.501	2.841-25.438	0.001
**Histologic grade**	I,II	1		0.800
	III	1.118	0.375-2.130	
**Ki67**		1.004	0.983-1.025	0.722
**PD-L1 expression**	Negative	1		0.038
	Positive	0.218	0.051-0.919	
		**Multivariable analysis**		
		**Hazard ratio**	**95% CI**	**P-value**
**PD-L1 expression**				0.0863
	Negative	1		
	Positive	0.280	0.065-1.120	
**Stage**				
	I	1		
	II	1.564	0.553-4.421	0.399
	III	6.886	2.278-20.811	0.001

IDC, invasive ductal carcinoma
