# Supplementary material for: Clinical and genomic assessment of PD-L1 SP142 expression in triple-negative breast cancer
Source: Breast Cancer Res Treat. 2021 Mar 26;188(1):165–78. doi: 10.1007/s10549-021-06193-9 (PMC8233296; doi:10.1007/s10549-021-06193-9)
Supplement: Supplementary file 7 — Supplementary file7 (PDF 115 kb) [file 10549_2021_6193_MOESM7_ESM.pdf]

Supplementary Table S3. Gene signatures, which were enriched in the SP142-top-two TNBC compared to the bottom-one in TNBC-TCGA

Gene signatures	Score(d)	Numerator(r)	Denominator(s+s0)	Fold Change	q-value(%)
TCGA.BRCA.1198_immune_CD19_Cell.2015_PMID.TBD	9.587059126	2.857251827	0.298032148	7.246336622	0
B_Cell_cluster_Iglesia_CCR.2014_PMID.24916698	10.20124868	2.832728783	0.277684514	7.124203779	0
TCGA.BRCA.1198_IMMUNE1_Cell.2015_PMID.TBD	10.17095054	2.770013386	0.272345576	6.821142425	0
T_Cell_cluster_Iglesia_CCR.2014_PMID.24916698	13.87912105	2.601507526	0.187440366	6.069204895	0
CD8_cluster_Iglesia_CCR.2014_PMID.24916698	13.49421216	2.595494897	0.192341344	6.043963287	0
TCGA.BRCA.1198_immune_CD8_GZMK_Cell.2015_PMID.TBD	13.47048217	2.500128163	0.185600495	5.657356802	0
TCGA.BRCA.1198_immune_CTLA4_CXCL_FOXP3_Cell.2015_PMID.TBD	14.45821258	2.36251889	0.163403248	5.142674668	0
Durvalumab_signature_Higgs_Clin.Cancer.Res.2018_PMID.29716923	11.98406013	2.213720635	0.18472209	4.63870029	0
MCD3_CD8_BMC.Med.Genomics.2011_PMID.21214954	12.51943913	2.13884551	0.170841959	4.404094758	0
LCK_Breast.Cancer.Research.2008_PMID.19272155	13.49842227	2.133703641	0.158070595	4.388426184	0
STAT1_Breast.Cancer.Research.2008_PMID.19272155	12.89349088	2.110137547	0.163659134	4.317324541	0
CIBERSORT_T_cells_CD8_Nat.Methods.2015_PMID.25822800	12.9015477	2.094416435	0.162338386	4.270533866	0
