# Supplementary material for: Clinical and genomic assessment of PD-L1 SP142 expression in triple-negative breast cancer
Source: Breast Cancer Res Treat. 2021 Mar 26;188(1):165–78. doi: 10.1007/s10549-021-06193-9 (PMC8233296; doi:10.1007/s10549-021-06193-9)
Supplement: Supplementary file 8 — Supplementary file8 (PDF 209 kb) [file 10549_2021_6193_MOESM8_ESM.pdf]

Supplementary Table S4. Melanoma cohort

A. Treatments of immune-check point inhibitors in melanoma cohorts

	Drugs	Number
**Anti-PD-1**	Pembrolizumab	32
	Nivolumab	9
**Combination blockade of PD-1 and CTLA-4**	Pembrolizumab +ipilimumab	25
	Nivolumab+ipilimumab	7

B. Continuous SP142 signature score for progression-free survival and overall survival in melanoma cohort

	HR	95% CI	P-value
**Progression-free survival**	0.604	0.472-0.773	6.26e-05
**Overall survival**	0.595	0.449-0.790	0.000326
